# The analytic network process for the pharmaceutical sector: Multi criteria decision making to select the suitable method for the preparation of nanoparticles

**DOI:** 10.1186/2008-2231-20-59

**Published:** 2012-10-18

**Authors:** Ramaiyan Velmurugan, Subramanian Selvamuthukumar

**Affiliations:** 1 Department of Pharmacy, Annamalai University, Annamalai nagar, 608002, Chidambaram, Tamilnadu, India; 2 Department of Pharmacetuical Technology, Annamalai University, Annamalai nagar, 608002, Tamilnadu, India

**Keywords:** Analytic network process, Multi criteria analysis, Decision analysis, Nanoparticles, Weighted matrix

## Abstract

**Background:**

This paper presents the methodology for assessing and selecting the most appropriate procedure for the preparation of nanoparticles by implementing the analytical network process. The commonly utilized nanoparticle preparation methods are Polymer Precipitation, Interfacial polymer deposition, Complex Coacervation, Cross linking, Emulsion solvent diffusion, Homogenization and Polymerization method. There are numerous parameters to be considered in groundwork of nanoparticles that departs the conclusion manufacturer in bias. One has to address a number of components in alignment to determine and choose the optimum conclusion choices, because an unsuitable conclusion could lead to the eventual merchandise having to be formulated and developed again. For this cause in this paper, we study selecting the most appropriate procedure for the preparation of nanoparticles utilizing one of the multi criteria-decision making techniques, Analytic Network Process.

**Methodology:**

The main goal was determined. The criteria and sub-criteria that affect the main goal were determined. The alternatives for the problem were determined. The interactions between criteria, sub-criteria, and alternatives respect to the main goal were determined. The super matrixes according to the network were assembled and then weighted super matrix and limit super matrix were then constructed. The values of this limit matrix are the desired priorities of the elements with respect to the goal. The alterative with the highest priority was finally chosen as the best alternative.

**Results:**

The emulsion solvent diffusion technique (M-5) has the highest value (0.434379) among the alternative methods that are applicable to the preparation of the nanoparticles. The second highest is Polymer Precipitation (M-1) with a value of 0.178798, and the lowest value or last choice is Cross Linking (M-4) with a value of only 0.024516. The alternative with the highest priority would achieve the goal, i.e., the best method for the preparation of the nanoparticles.

**Conclusion:**

The alternative M5 emulsion solvent diffusion technique, scoring 0.434379 was the one with largest main concern amidst all the other alternatives and thereby judged to be the most apt procedure for the preparation of nanoparticles.

## Background

Discovery and development of drugs for the remedy of diseases are a multidisciplinary locality that requires extends commitment to conduct technical research. Latest studies disclosed that the mean time to discover, develop and approve a new pharmaceutical in the united states takes roughly 14.2 years [1] with an approximated research expenditure of $800-$900 million in 2003 [2-4] and a separate analysis estimated the cost at $ 1.3-$1.6 billion in 2005 [5]. In the last few decades, Pharmaceutical formulations with Novel Drug Delivery System (NDDS) have been presented with the aim of optimizing bioavailability by modulating the time course of the drug concentration in body-fluid [6,7]. Novel drug-delivery systems (NDDS) are amidst the localities at the cutting brim of study in the pharmaceutical area today. All sustained and controlled release products have the widespread aim of advancing drug treatment over that accomplished with their non-sustained and non-controlled release equivalent [8,9].

The absorption rate of a drug can be declined by decreasing its rate of release from the dosage form. The merchandise so formulated is conceived as a sustained action, sustained release, prolonged action, depot, retarded release, delayed action or timed release medication [10]. This latest interest has been due to diverse factors, viz: the prohibitive cost of developing new drug entities, expiry of worldwide patents, discovery of new polymeric materials suitable for extending drug release, and improvements in therapeutic efficacy and safety accomplished by these consignment schemes [11,12]. Various approaches are accessible for accomplishing NDDS such as targeted delivery schemes, nanoparticles, prodrugs, transdermal schemes, ocular schemes, intravaginal and intrauterine schemes, injections and implants, microencapsulation, matrix devices, and reservoir devices. One of the most productive advances comprised nanoparticles.

A number of techniques are available for the groundwork of nanoparticles that encompass Polymerization, Interfacial complexation, Emulsion chemical dehydration, Desolvation or phase separation, pH induced aggregation, Solvent extraction procedures, Double emulsion solvent evaporation method, Salting out, Solvent displacement, nano precipitation, Solvent diffusion, Polymer precipitation, Complex coacervation, cross linking and homogenization methods [13-15].

The picture of pharmaceutical development being like this, a serious approach has to be concentrated on the fine and an unquestionable decision-making and finalizing the decisions.

While choosing technique, concern of the cost factors alone may not be justifiable. It is more reasonable and befitting to analyze both qualitative and quantitative parameters and then to make a conclusion when two or more options are in hand and one has to choose the best. Further, when there are such many parameters to be advised in preparation of nanoparticles, the conclusion maker has to consider a number of components in alignment to work out and choose the optimum decision choices, because an unsuitable conclusion could lead to the eventual merchandise having to be formulated and developed again.

Multi-Criteria decision-making is a well-known agency of decision making. It is an agency of a general class of operation's research models, which deal with conclusion troubles in the occurrence of a number of conclusion criteria. This major class of models is very often called MCDM. This class is further divided into multi objective decision making (MODM) and multi-attribute decision making (MADM) [16]. There are some methods in each of the overhead classes. Priority founded, outranking, expanse founded and blended methods are also directed to diverse troubles. Each procedure has its own characteristics, and the procedures can furthermore be classified as deterministic, stochastic and fuzzy procedures. There may be combinations of the overhead procedures [17,18].

In this paper, we deal with one of the preparation procedure assortment problems, selecting an apt and an appropriate preparation procedure for the preparation of the nanoparticles, using one of the multi criteria-decision making methods, Analytic Network Process (ANP), because this problem has some criteria and some options for decision-making as asserted above. The rest of this paper is organized as pursues: a short introduction to ANP, an application for appropriate preparation procedure assortment utilizing ANP is offered. The last part summarizes the outcome and makes proposals for further research.

### Analytic network process

Choosing or prioritizing alternatives from a set of accessible alternatives with respect to multiple criteria, is often referred to Multi-Criteria Decision Making (MCDM). Analytic Hierarchy Process (AHP) and Analytic Network Process (ANP) are the common methods by which to solve Multi-Criteria Decision Making difficulties. The decision difficulty is structured hierarchically at distinct grades in both methodologies. The local priorities concerns in ANP are established in the identical kind as they are in AHP utilizing pair-wise comparisons and judgments [19,20]. The Analytic Network Process is the generalization of Saaty’s Analytic Hierarchy Process, which is one of the most widely, engaged decision support devices [21]. Likewise, to the AHP, the priorities in the ANP are assessed obscurely from pair-wise comparisons judgments [22]. There are four general steps in ANP founded multicriteria decision-making method, including model construction; paired assessments between each two cluster or nodes; super matrix assessment based on outcomes from paired comparisons; and result investigation for the evaluation [23,24].

For the proposed ANP algorithm, the steps are as shown below:

• Step 1: Analyse the problem, and determine the main goal.

• Step 2: Determine the criteria and sub-criteria that affect the main goal.

• Step 3: Determine alternatives for the problem.

• Step 4: Determine the interactions between criteria, sub-criteria, and alternative's respect to the main goal.

• Step 5: Construct a super matrix according to the network, and then construct weighted super matrix and limit super matrix. In a super matrix, each element is represented at one row and one respective column. If the column sum of any column in the composed super matrix is greater than 1, that column will be normalized. Such a super matrix is called as the weighted super matrix. The weighted super matrix is then raised to a significantly large power in order to have the converged or stable values. The values of this limit matrix are the desired priorities of the elements with respect to the goal [25].

• Step 6: Choose the best alternative with the highest priority.

In the literature, ANP method has been used to solve problems like Research and Development Project Selection [26], Performance Evaluation [27], Quality Function Deployment Implementation [28], Enterprise Resource Planning (ERP) Software Selection [29]. In this paper, Saaty’s ANP [30] is used for the pharmaceutical sector in selecting an appropriate method for the preparation of nanoparticles.

## Methods

### Nanoparticle preparation method selection using analytic network process

**Figure 1:**
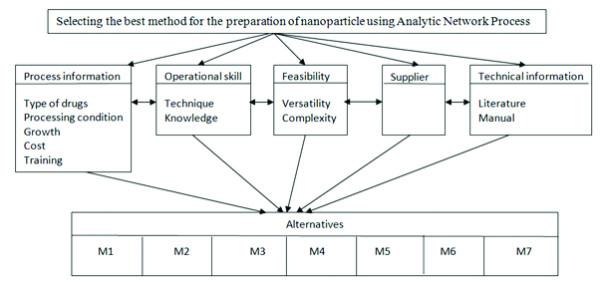
The model for the problem, selecting the most appropriate method using ANP.

For the numerical example in this study, preparation method selection problem has chosen and for this problem ANP approach has used. For the production of the nanoparticles, an appropriate method is in need where the selected method should be feasible and should be in a way to achieve reproducibility and consistency with good entrapment efficiency, etc. For this problem decision criterion and alternatives have defined with experts as seen on Figure 1. In this paper, main criteria are process information, operational skill, feasibility, supplier and technical information.

### Process information criteria

This main criterion includes the sub-criteria explaining about processing details. Sub-criteria under this title includes “Types of drugs,” “Processing condition,” “Growth in the field,” “Cost” and “Training”.

### Operational skill criteria

Under these criteria, there are these sub-criteria; “Technique” and “Knowledge” about the method.

### Feasibility criteria

Under the feasibility criteria, these sub-criteria can be thought; “Versatility” and “Complexity.”

### Technical information criteria

This would include the sub criteria, “Literature” and “Manual”.

**Figure 2:**
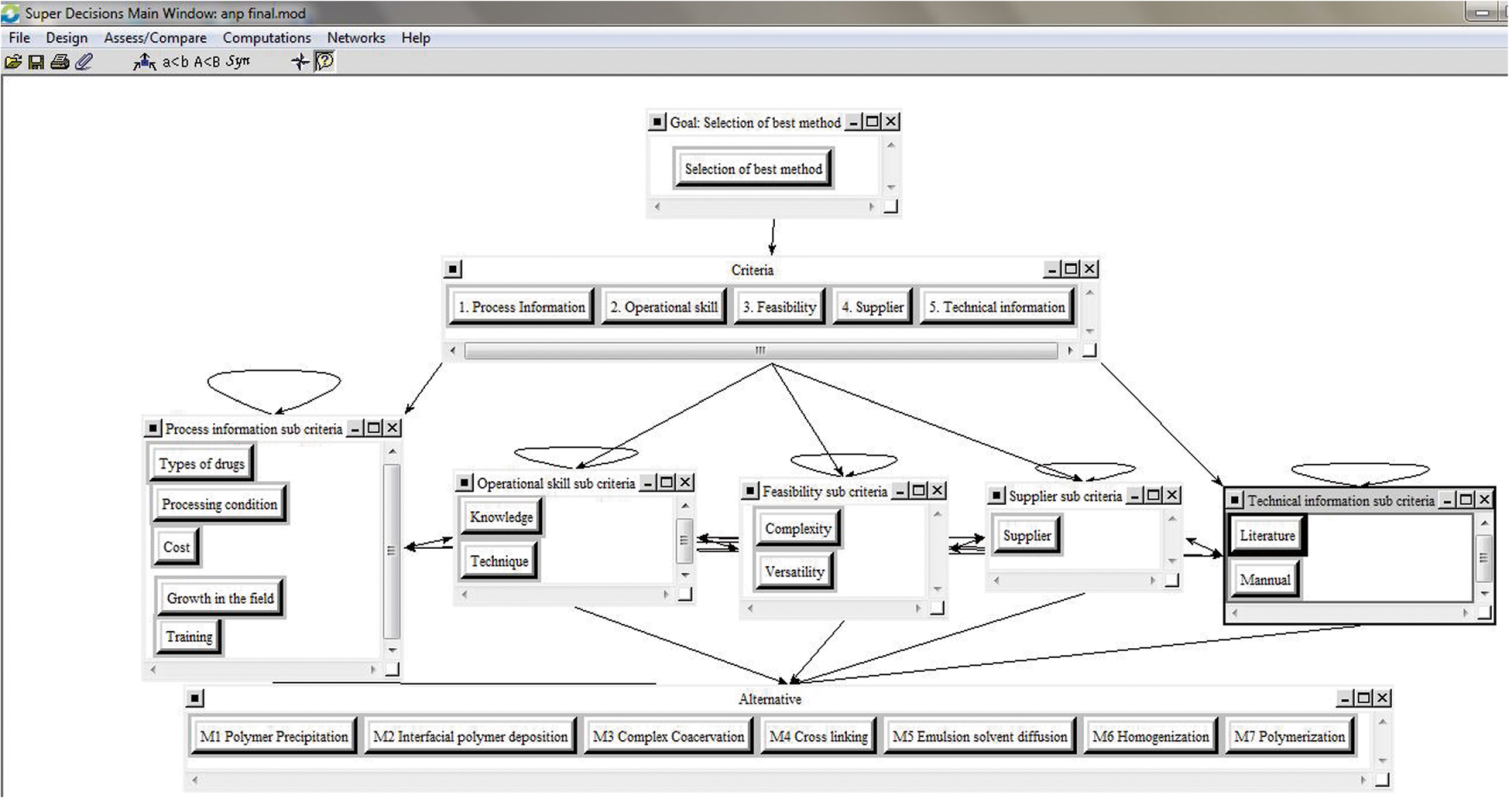
The Relation between Goal, Criteria, Sub-Criteria and the Alternatives.

As seen on Figure 1 and Figure 2, the alternatives for preparation method selection are M1 Polymer precipitation, M2 Interfacial polymer deposition, M3 Complex coacervation, M4 Cross linking, M5 Emulsion solvent diffusion, M6 Homogenization and M7 Polymerization technique. In Figure 1 the model that written to Super Decisions software can be seen.

**Figure 3:**
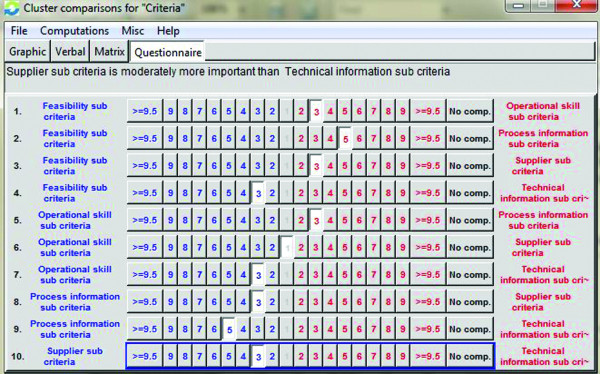
Entering data to super decision software.

After the purpose, criteria and alternatives have determined, binary comparisons have done. After all of binary comparisons have completed, these comparison's data are entered to Super Decisions software which is illustrated in Figure 3.

The super matrix used in this paper is as follows:

Where w1 is a vector that represents the impact of the goal on the criteria (CRs). W2 is a matrix that denotes the impact of the CRs on each of the alternatives (ALTs), W3 and W4 are the matrices that represent the inner dependence of the CRs and the inner dependence of the

(1) $$\begin{array}{l} \;Goal\left(G\right)CRsALTs\\ \begin{array}{c} Goal\left(G\right)\\ W=CRs\\ ALTs \end{array}\left[\begin{array}{ccc} 0 & 0 & 0\\ W_{1} & W_{3} & 0\\ 0 & W_{2} & W_{4} \end{array}\right] \end{array}$$

ALTs, respectively. After all of data have entered the program, lastly the result can be found. For our problem, the unweighted super matrix, weighted super matrix and limit super matrix are shown in Tables 1, 2, and 3, respectively.

**Table 1:** Unweighted matrix

	**M1**	**M2**	**M3**	**M4**	**M5**	**M6**	**M7**	**PI**	**OS**	**FE**	**SU**	**TE**	**CM**	**VE**	**GOAL**	**KN**	**TE**	**CO**	**GR**	**PC**	**TR**	**TD**	**SU**	**LT**	**MN**
M1	0.00000	0.00000	0.00000	0.00000	0.00000	0.00000	0.00000	0.00000	0.00000	0.00000	0.00000	0.00000	0.14286	0.00000	0.00000	0.14286	0.00000	0.00000	0.00000	0.00000	0.00000	0.17880	0.14286	0.14286	0.00000
M2	0.00000	0.00000	0.00000	0.00000	0.00000	0.00000	0.00000	0.00000	0.00000	0.00000	0.00000	0.00000	0.14286	0.00000	0.00000	0.14286	0.00000	0.00000	0.00000	0.00000	0.00000	0.05395	0.14286	0.14286	0.00000
M3	0.00000	0.00000	0.00000	0.00000	0.00000	0.00000	0.00000	0.00000	0.00000	0.00000	0.00000	0.00000	0.14286	0.00000	0.00000	0.14286	0.00000	0.00000	0.00000	0.00000	0.00000	0.07430	0.14286	0.14286	0.00000
M4	0.00000	0.00000	0.00000	0.00000	0.00000	0.00000	0.00000	0.00000	0.00000	0.00000	0.00000	0.00000	0.14286	0.00000	0.00000	0.14286	0.00000	0.00000	0.00000	0.00000	0.00000	0.02452	0.14286	0.14286	0.00000
M5	0.00000	0.00000	0.00000	0.00000	0.00000	0.00000	0.00000	0.00000	0.00000	0.00000	0.00000	0.00000	0.14286	0.00000	0.00000	0.14286	0.00000	0.00000	0.00000	0.00000	0.00000	0.43438	0.14286	0.14286	0.00000
M6	0.00000	0.00000	0.00000	0.00000	0.00000	0.00000	0.00000	0.00000	0.00000	0.00000	0.00000	0.00000	0.14286	0.00000	0.00000	0.14286	0.00000	0.00000	0.00000	0.00000	0.00000	0.09887	0.14286	0.14286	0.00000
M7	0.00000	0.00000	0.00000	0.00000	0.00000	0.00000	0.00000	0.00000	0.00000	0.00000	0.00000	0.00000	0.14286	0.00000	0.00000	0.14286	0.00000	0.00000	0.00000	0.00000	0.00000	0.13518	0.14286	0.14286	0.00000
PI	0.00000	0.00000	0.00000	0.00000	0.00000	0.00000	0.00000	0.00000	0.00000	0.00000	0.00000	0.00000	0.00000	0.00000	0.00000	0.00000	0.00000	0.00000	0.00000	0.00000	0.00000	0.00000	0.00000	0.00000	0.00000
OS	0.00000	0.00000	0.00000	0.00000	0.00000	0.00000	0.00000	0.00000	0.00000	0.00000	0.00000	0.00000	0.00000	0.00000	0.00000	0.00000	0.00000	0.00000	0.00000	0.00000	0.00000	0.00000	0.00000	0.00000	0.00000
FE	0.00000	0.00000	0.00000	0.00000	0.00000	0.00000	0.00000	0.00000	0.00000	0.00000	0.00000	0.00000	0.00000	0.00000	0.00000	0.00000	0.00000	0.00000	0.00000	0.00000	0.00000	0.00000	0.00000	0.00000	0.00000
SU	0.00000	0.00000	0.00000	0.00000	0.00000	0.00000	0.00000	0.00000	0.00000	0.00000	0.00000	0.00000	0.00000	0.00000	0.00000	0.00000	0.00000	0.00000	0.00000	0.00000	0.00000	0.00000	0.00000	0.00000	0.00000
TE	0.00000	0.00000	0.00000	0.00000	0.00000	0.00000	0.00000	0.00000	0.00000	0.00000	0.00000	0.00000	0.00000	0.00000	0.00000	0.00000	0.00000	0.00000	0.00000	0.00000	0.00000	0.00000	0.00000	0.00000	0.00000
CM	0.00000	0.00000	0.00000	0.00000	0.00000	0.00000	0.00000	0.24998	0.00000	0.00000	0.00000	0.00000	0.24998	0.00000	0.00000	0.50000	0.00000	0.00000	0.00000	0.00000	0.00000	1.00000	0.50000	0.50000	0.00000
VE	0.00000	0.00000	0.00000	0.00000	0.00000	0.00000	0.00000	0.75002	0.00000	0.00000	0.00000	0.00000	0.75002	0.00000	0.00000	0.50000	0.00000	0.00000	0.00000	0.00000	0.00000	0.00000	0.50000	0.50000	0.00000
GOAL	0.00000	0.00000	0.00000	0.00000	0.00000	0.00000	0.00000	0.00000	0.00000	0.00000	0.00000	0.00000	0.00000	0.00000	0.00000	0.00000	0.00000	0.00000	0.00000	0.00000	0.00000	0.00000	0.00000	0.00000	0.00000
KN	0.00000	0.00000	0.00000	0.00000	0.00000	0.00000	0.00000	0.24998	0.00000	0.00000	0.00000	0.00000	0.50000	0.00000	0.00000	0.50000	0.00000	0.00000	0.00000	0.00000	0.00000	0.50000	0.50000	0.50000	0.00000
TE	0.00000	0.00000	0.00000	0.00000	0.00000	0.00000	0.00000	0.75002	0.00000	0.00000	0.00000	0.00000	0.50000	0.00000	0.00000	0.50000	0.00000	0.00000	0.00000	0.00000	0.00000	0.50000	0.50000	0.50000	0.00000
CO	0.00000	0.00000	0.00000	0.00000	0.00000	0.00000	0.00000	0.13631	0.00000	0.00000	0.00000	0.00000	0.20000	0.00000	0.00000	0.20000	0.00000	0.00000	0.00000	0.00000	0.00000	0.33333	0.20000	0.20000	0.00000
GR	0.00000	0.00000	0.00000	0.00000	0.00000	0.00000	0.00000	0.06805	0.00000	0.00000	0.00000	0.00000	0.20000	0.00000	0.00000	0.20000	0.00000	0.00000	0.00000	0.00000	0.00000	0.33333	0.20000	0.20000	0.00000
PC	0.00000	0.00000	0.00000	0.00000	0.00000	0.00000	0.00000	0.26324	0.00000	0.00000	0.00000	0.00000	0.20000	0.00000	0.00000	0.20000	0.00000	0.00000	0.00000	0.00000	0.00000	0.33333	0.20000	0.20000	0.00000
TR	0.00000	0.00000	0.00000	0.00000	0.00000	0.00000	0.00000	0.12006	0.00000	0.00000	0.00000	0.00000	0.20000	0.00000	0.00000	0.20000	0.00000	0.00000	0.00000	0.00000	0.00000	0.00000	0.20000	0.20000	0.00000
TD	0.00000	0.00000	0.00000	0.00000	0.00000	0.00000	0.00000	0.39234	0.00000	0.00000	0.00000	0.00000	0.20000	0.00000	0.00000	0.20000	0.00000	0.00000	0.00000	0.00000	0.00000	0.00000	0.20000	0.20000	0.00000
SU	0.00000	0.00000	0.00000	0.00000	0.00000	0.00000	0.00000	1.00000	0.00000	0.00000	0.00000	0.00000	1.00000	0.00000	0.00000	1.00000	0.00000	0.00000	0.00000	0.00000	0.00000	1.00000	1.00000	1.00000	0.00000
LT	0.00000	0.00000	0.00000	0.00000	0.00000	0.00000	0.00000	0.75000	0.00000	0.00000	0.00000	0.00000	0.50000	0.00000	0.00000	0.50000	0.00000	0.00000	0.00000	0.00000	0.00000	0.50000	0.50000	0.50000	0.00000
MN	0.00000	0.00000	0.00000	0.00000	0.00000	0.00000	0.00000	0.25000	0.00000	0.00000	0.00000	0.00000	0.50000	0.00000	0.00000	0.50000	0.00000	0.00000	0.00000	0.00000	0.00000	0.50000	0.50000	0.50000	0.00000

**Table 2:** Weighted matrix

	**M1**	**M2**	**M3**	**M4**	**M5**	**M6**	**M7**	**PI**	**OS**	**FE**	**SU**	**TE**	**CM**	**VE**	**GOAL**	**KN**	**TE**	**CO**	**GR**	**PC**	**TR**	**TD**	**SU**	**LT**	**MN**
M1	0.00000	0.00000	0.00000	0.00000	0.00000	0.00000	0.00000	0.00000	0.00000	0.00000	0.00000	0.00000	0.02381	0.00000	0.00000	0.02381	0.00000	0.00000	0.00000	0.00000	0.00000	0.02980	0.02381	0.02381	0.00000
M2	0.00000	0.00000	0.00000	0.00000	0.00000	0.00000	0.00000	0.00000	0.00000	0.00000	0.00000	0.00000	0.02381	0.00000	0.00000	0.02381	0.00000	0.00000	0.00000	0.00000	0.00000	0.00699	0.02381	0.02381	0.00000
M3	0.00000	0.00000	0.00000	0.00000	0.00000	0.00000	0.00000	0.00000	0.00000	0.00000	0.00000	0.00000	0.02381	0.00000	0.00000	0.02381	0.00000	0.00000	0.00000	0.00000	0.00000	0.01238	0.02381	0.02381	0.00000
M4	0.00000	0.00000	0.00000	0.00000	0.00000	0.00000	0.00000	0.00000	0.00000	0.00000	0.00000	0.00000	0.02381	0.00000	0.00000	0.02381	0.00000	0.00000	0.00000	0.00000	0.00000	0.00409	0.02381	0.02381	0.00000
M5	0.00000	0.00000	0.00000	0.00000	0.00000	0.00000	0.00000	0.00000	0.00000	0.00000	0.00000	0.00000	0.02381	0.00000	0.00000	0.02381	0.00000	0.00000	0.00000	0.00000	0.00000	0.07240	0.02381	0.02381	0.00000
M6	0.00000	0.00000	0.00000	0.00000	0.00000	0.00000	0.00000	0.00000	0.00000	0.00000	0.00000	0.00000	0.02381	0.00000	0.00000	0.02381	0.00000	0.00000	0.00000	0.00000	0.00000	0.01648	0.02381	0.02381	0.00000
M7	0.00000	0.00000	0.00000	0.00000	0.00000	0.00000	0.00000	0.00000	0.00000	0.00000	0.00000	0.00000	0.02381	0.00000	0.00000	0.02381	0.00000	0.00000	0.00000	0.00000	0.00000	0.02253	0.02381	0.02381	0.00000
PI	0.00000	0.00000	0.00000	0.00000	0.00000	0.00000	0.00000	0.00000	0.00000	0.00000	0.00000	0.00000	0.00000	0.00000	0.00000	0.00000	0.00000	0.00000	0.00000	0.00000	0.00000	0.00000	0.00000	0.00000	0.00000
OS	0.00000	0.00000	0.00000	0.00000	0.00000	0.00000	0.00000	0.00000	0.00000	0.00000	0.00000	0.00000	0.00000	0.00000	0.00000	0.00000	0.00000	0.00000	0.00000	0.00000	0.00000	0.00000	0.00000	0.00000	0.00000
FE	0.00000	0.00000	0.00000	0.00000	0.00000	0.00000	0.00000	0.00000	0.00000	0.00000	0.00000	0.00000	0.00000	0.00000	0.00000	0.00000	0.00000	0.00000	0.00000	0.00000	0.00000	0.00000	0.00000	0.00000	0.00000
SU	0.00000	0.00000	0.00000	0.00000	0.00000	0.00000	0.00000	0.00000	0.00000	0.00000	0.00000	0.00000	0.00000	0.00000	0.00000	0.00000	0.00000	0.00000	0.00000	0.00000	0.00000	0.00000	0.00000	0.00000	0.00000
TE	0.00000	0.00000	0.00000	0.00000	0.00000	0.00000	0.00000	0.00000	0.00000	0.00000	0.00000	0.00000	0.00000	0.00000	0.00000	0.00000	0.00000	0.00000	0.00000	0.00000	0.00000	0.00000	0.00000	0.00000	0.00000
CM	0.00000	0.00000	0.00000	0.00000	0.00000	0.00000	0.00000	0.02378	0.00000	0.00000	0.00000	0.00000	0.06333	0.00000	0.00000	0.06333	0.00000	0.00000	0.00000	0.00000	0.00000	0.16667	0.06333	0.06333	0.00000
VE	0.00000	0.00000	0.00000	0.00000	0.00000	0.00000	0.00000	0.07135	0.00000	0.00000	0.00000	0.00000	0.06333	0.00000	0.00000	0.06333	0.00000	0.00000	0.00000	0.00000	0.00000	0.00000	0.06333	0.06333	0.00000
GOAL	0.00000	0.00000	0.00000	0.00000	0.00000	0.00000	0.00000	0.00000	0.00000	0.00000	0.00000	0.00000	0.00000	0.00000	0.00000	0.00000	0.00000	0.00000	0.00000	0.00000	0.00000	0.00000	0.00000	0.00000	0.00000
KN	0.00000	0.00000	0.00000	0.00000	0.00000	0.00000	0.00000	0.04820	0.00000	0.00000	0.00000	0.00000	0.06333	0.00000	0.00000	0.06333	0.00000	0.00000	0.00000	0.00000	0.00000	0.06333	0.06333	0.06333	0.00000
TE	0.00000	0.00000	0.00000	0.00000	0.00000	0.00000	0.00000	0.14462	0.00000	0.00000	0.00000	0.00000	0.06333	0.00000	0.00000	0.06333	0.00000	0.00000	0.00000	0.00000	0.00000	0.06333	0.06333	0.06333	0.00000
CO	0.00000	0.00000	0.00000	0.00000	0.00000	0.00000	0.00000	0.06261	0.00000	0.00000	0.00000	0.00000	0.03333	0.00000	0.00000	0.03333	0.00000	0.00000	0.00000	0.00000	0.00000	0.05556	0.03333	0.03333	0.00000
GR	0.00000	0.00000	0.00000	0.00000	0.00000	0.00000	0.00000	0.04044	0.00000	0.00000	0.00000	0.00000	0.03333	0.00000	0.00000	0.03333	0.00000	0.00000	0.00000	0.00000	0.00000	0.05556	0.03333	0.03333	0.00000
PC	0.00000	0.00000	0.00000	0.00000	0.00000	0.00000	0.00000	0.12091	0.00000	0.00000	0.00000	0.00000	0.03333	0.00000	0.00000	0.03333	0.00000	0.00000	0.00000	0.00000	0.00000	0.05556	0.03333	0.03333	0.00000
TR	0.00000	0.00000	0.00000	0.00000	0.00000	0.00000	0.00000	0.05515	0.00000	0.00000	0.00000	0.00000	0.03333	0.00000	0.00000	0.03333	0.00000	0.00000	0.00000	0.00000	0.00000	0.00000	0.03333	0.03333	0.00000
TD	0.00000	0.00000	0.00000	0.00000	0.00000	0.00000	0.00000	0.18021	0.00000	0.00000	0.00000	0.00000	0.03333	0.00000	0.00000	0.03333	0.00000	0.00000	0.00000	0.00000	0.00000	0.00000	0.03333	0.03333	0.00000
SU	0.00000	0.00000	0.00000	0.00000	0.00000	0.00000	0.00000	0.19281	0.00000	0.00000	0.00000	0.00000	0.16667	0.00000	0.00000	0.16667	0.00000	0.00000	0.00000	0.00000	0.00000	0.16667	0.16667	0.16667	0.00000
LT	0.00000	0.00000	0.00000	0.00000	0.00000	0.00000	0.00000	0.04495	0.00000	0.00000	0.00000	0.00000	0.06333	0.00000	0.00000	0.06333	0.00000	0.00000	0.00000	0.00000	0.00000	0.06333	0.06333	0.06333	0.00000
MN	0.00000	0.00000	0.00000	0.00000	0.00000	0.00000	0.00000	0.01495	0.00000	0.00000	0.00000	0.00000	0.06333	0.00000	0.00000	0.06333	0.00000	0.00000	0.00000	0.00000	0.00000	0.06333	0.06333	0.06333	0.00000

**Table 3:** Limit matrix

	**M1**	**M2**	**M3**	**M4**	**M5**	**M6**	**M7**	**PI**	**OS**	**FE**	**SU**	**TE**	**CM**	**VE**	**GOAL**	**KN**	**TE**	**CO**	**GR**	**PC**	**TR**	**TD**	**SU**	**LT**	**MN**
M1	0.00000	0.00000	0.00000	0.00000	0.00000	0.00000	0.00000	0.02423	0.00000	0.00000	0.00000	0.00000	0.02423	0.00000	0.02423	0.02423	0.00000	0.00000	0.00000	0.00000	0.00000	0.02423	0.02423	0.02423	0.00000
M2	0.00000	0.00000	0.00000	0.00000	0.00000	0.00000	0.00000	0.02278	0.00000	0.00000	0.00000	0.00000	0.02278	0.00000	0.02278	0.02278	0.00000	0.00000	0.00000	0.00000	0.00000	0.02278	0.02278	0.02278	0.00000
M3	0.00000	0.00000	0.00000	0.00000	0.00000	0.00000	0.00000	0.02302	0.00000	0.00000	0.00000	0.00000	0.02302	0.00000	0.02302	0.02302	0.00000	0.00000	0.00000	0.00000	0.00000	0.02302	0.02302	0.02302	0.00000
M4	0.00000	0.00000	0.00000	0.00000	0.00000	0.00000	0.00000	0.02244	0.00000	0.00000	0.00000	0.00000	0.02244	0.00000	0.02244	0.02244	0.00000	0.00000	0.00000	0.00000	0.00000	0.02244	0.02244	0.02244	0.00000
M5	0.00000	0.00000	0.00000	0.00000	0.00000	0.00000	0.00000	0.02719	0.00000	0.00000	0.00000	0.00000	0.02719	0.00000	0.02719	0.02719	0.00000	0.00000	0.00000	0.00000	0.00000	0.02719	0.02719	0.02719	0.00000
M6	0.00000	0.00000	0.00000	0.00000	0.00000	0.00000	0.00000	0.02330	0.00000	0.00000	0.00000	0.00000	0.02330	0.00000	0.02330	0.02330	0.00000	0.00000	0.00000	0.00000	0.00000	0.02330	0.02330	0.02330	0.00000
M7	0.00000	0.00000	0.00000	0.00000	0.00000	0.00000	0.00000	0.02372	0.00000	0.00000	0.00000	0.00000	0.02372	0.00000	0.02372	0.02372	0.00000	0.00000	0.00000	0.00000	0.00000	0.02372	0.02372	0.02372	0.00000
PI	0.00000	0.00000	0.00000	0.00000	0.00000	0.00000	0.00000	0.00000	0.00000	0.00000	0.00000	0.00000	0.00000	0.00000	0.00000	0.00000	0.00000	0.00000	0.00000	0.00000	0.00000	0.00000	0.00000	0.00000	0.00000
OS	0.00000	0.00000	0.00000	0.00000	0.00000	0.00000	0.00000	0.00000	0.00000	0.00000	0.00000	0.00000	0.00000	0.00000	0.00000	0.00000	0.00000	0.00000	0.00000	0.00000	0.00000	0.00000	0.00000	0.00000	0.00000
FE	0.00000	0.00000	0.00000	0.00000	0.00000	0.00000	0.00000	0.00000	0.00000	0.00000	0.00000	0.00000	0.00000	0.00000	0.00000	0.00000	0.00000	0.00000	0.00000	0.00000	0.00000	0.00000	0.00000	0.00000	0.00000
SU	0.00000	0.00000	0.00000	0.00000	0.00000	0.00000	0.00000	0.00000	0.00000	0.00000	0.00000	0.00000	0.00000	0.00000	0.00000	0.00000	0.00000	0.00000	0.00000	0.00000	0.00000	0.00000	0.00000	0.00000	0.00000
TE	0.00000	0.00000	0.00000	0.00000	0.00000	0.00000	0.00000	0.00000	0.00000	0.00000	0.00000	0.00000	0.00000	0.00000	0.00000	0.00000	0.00000	0.00000	0.00000	0.00000	0.00000	0.00000	0.00000	0.00000	0.00000
CM	0.00000	0.00000	0.00000	0.00000	0.00000	0.00000	0.00000	0.06151	0.00000	0.00000	0.00000	0.00000	0.06151	0.00000	0.06151	0.06151	0.00000	0.00000	0.00000	0.00000	0.00000	0.06151	0.06151	0.06151	0.00000
VE	0.00000	0.00000	0.00000	0.00000	0.00000	0.00000	0.00000	0.06515	0.00000	0.00000	0.00000	0.00000	0.06515	0.00000	0.06515	0.06515	0.00000	0.00000	0.00000	0.00000	0.00000	0.06515	0.06515	0.06515	0.00000
GOAL	0.00000	0.00000	0.00000	0.00000	0.00000	0.00000	0.00000	0.00000	0.00000	0.00000	0.00000	0.00000	0.00000	0.00000	0.00000	0.00000	0.00000	0.00000	0.00000	0.00000	0.00000	0.00000	0.00000	0.00000	0.00000
KN	0.00000	0.00000	0.00000	0.00000	0.00000	0.00000	0.00000	0.06333	0.00000	0.00000	0.00000	0.00000	0.06333	0.00000	0.06333	0.06333	0.00000	0.00000	0.00000	0.00000	0.00000	0.06333	0.06333	0.06333	0.00000
TE	0.00000	0.00000	0.00000	0.00000	0.00000	0.00000	0.00000	0.06333	0.00000	0.00000	0.00000	0.00000	0.06333	0.00000	0.06333	0.06333	0.00000	0.00000	0.00000	0.00000	0.00000	0.06333	0.06333	0.06333	0.00000
CO	0.00000	0.00000	0.00000	0.00000	0.00000	0.00000	0.00000	0.03488	0.00000	0.00000	0.00000	0.00000	0.03488	0.00000	0.03488	0.03488	0.00000	0.00000	0.00000	0.00000	0.00000	0.03488	0.03488	0.03488	0.00000
GR	0.00000	0.00000	0.00000	0.00000	0.00000	0.00000	0.00000	0.03488	0.00000	0.00000	0.00000	0.00000	0.03488	0.00000	0.03488	0.03488	0.00000	0.00000	0.00000	0.00000	0.00000	0.03488	0.03488	0.03488	0.00000
PC	0.00000	0.00000	0.00000	0.00000	0.00000	0.00000	0.00000	0.03488	0.00000	0.00000	0.00000	0.00000	0.03488	0.00000	0.03488	0.03488	0.00000	0.00000	0.00000	0.00000	0.00000	0.03488	0.03488	0.03488	0.00000
TR	0.00000	0.00000	0.00000	0.00000	0.00000	0.00000	0.00000	0.03102	0.00000	0.00000	0.00000	0.00000	0.03102	0.00000	0.03102	0.03102	0.00000	0.00000	0.00000	0.00000	0.00000	0.03102	0.03102	0.03102	0.00000
TD	0.00000	0.00000	0.00000	0.00000	0.00000	0.00000	0.00000	0.03102	0.00000	0.00000	0.00000	0.00000	0.03102	0.00000	0.03102	0.03102	0.00000	0.00000	0.00000	0.00000	0.00000	0.03102	0.03102	0.03102	0.00000
SU	0.00000	0.00000	0.00000	0.00000	0.00000	0.00000	0.00000	0.16667	0.00000	0.00000	0.00000	0.00000	0.16667	0.00000	0.16667	0.16667	0.00000	0.00000	0.00000	0.00000	0.00000	0.16667	0.16667	0.16667	0.00000
LT	0.00000	0.00000	0.00000	0.00000	0.00000	0.00000	0.00000	0.06333	0.00000	0.00000	0.00000	0.00000	0.06333	0.00000	0.06333	0.06333	0.00000	0.00000	0.00000	0.00000	0.00000	0.06333	0.06333	0.06333	0.00000
MN	0.00000	0.00000	0.00000	0.00000	0.00000	0.00000	0.00000	0.06333	0.00000	0.00000	0.00000	0.00000	0.06333	0.00000	0.06333	0.06333	0.00000	0.00000	0.00000	0.00000	0.00000	0.06333	0.06333	0.06333	0.00000

**Figure 4:**
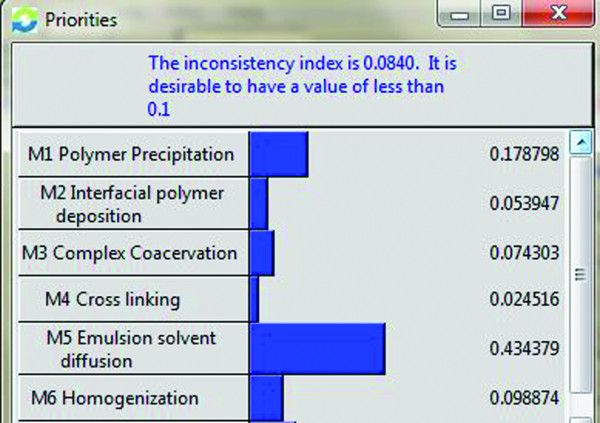
Overall Priority for the Alternatives.

Lastly, for our problem, the weights of selecting the best method for the preparation of the nanoparticle have been calculated as shown in Figure 4.

## Results and discussions

**Figure 5:**
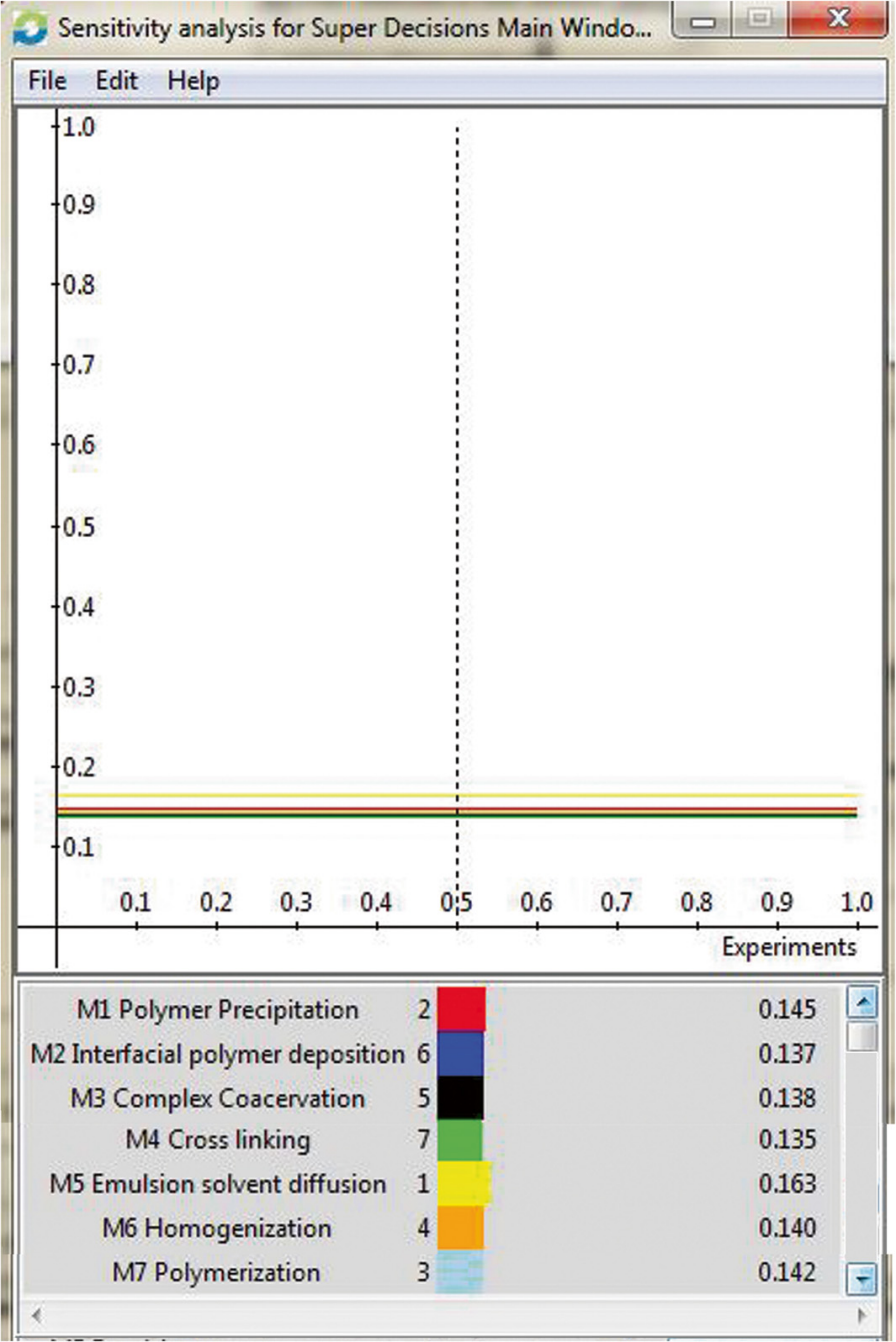
Sensitivity analysis.

Following results were got showing the priorities of the alternatives with esteem to criteria and sub criteria. With that of the general main concern of the criteria, processing information is given much significant pursued by operational skill and supplier which are then pursued by feasibility and cost. The general main concern of the alternatives M1 Polymer Precipitation, M2 Interfacial Polymer Deposition, M3 Complex Coacervation, M4 Cross Linking, M5 Emulsion Solvent Diffusion, M6 Homogenization and M7 Polymerization are shown in the Figure 4 asserting Method M5 is the best procedure on the basis of criteria and sub criteria pursued by M1, M7, M6, M3, M2 and M4. The sensitivity investigation of the decisions made is shown in the Figure 5 which gives a general representation of the peak criterion and sub criterion. The standards of the matrixes are the yearned priorities of the components with respect to the aim. The values of the unweighted matrix, weighted matrix and limit matrix are shown in Tables 1, 2 and 3 respectively displaying the priorities of the criteria and sub criteria with which the importance is judged.

Procedure assortment, which is one of the most significant steps in the formulation method, must be systematically advised by the decision manufacturers. For this cause, method assortment is assessed by us in this study work in a large framework consisting of diverse methods from the untested to the analytical ones and assessed a befitting procedure that would opt the preparation of nanoparticles. It is virtually an adversity to structure the hierarchy for numerous conclusion problems when the interaction of higher-level components with smaller grade components, and their dependency should be taken into account. The ANP supplies a solution for problems, which will not be organized hierarchically. Not only does the importance of the Criteria work out the significance of the alternatives, as in a hierarchy, but the significance of the alternatives themselves determines the significance of the criteria [31].

In this paper, procedure assortment was advised as a multi criteria decision difficulty and a form is suggested by using ANP. The evaluation criteria and sub criteria were developed according to the formulation scenario, and the form was directed to a genuine case study. This paper displays that, ANP is a decision device by making strategical conclusions, such as selecting a best method to arrange nanoparticles. Generally, conclusion manufacturers might be inclined not to use a sophisticated method, but by utilizing client amicable software like the super decision, evolved by Saaty, the decision-making method by utilizing ANP will be handled simpler. Assessing the best method from both objective and subjective criteria will gain flexibility to the conclusion process. When we believe, a method’s all purposeful components; all these components have relationships with the other ones, so we can effortlessly say that all the criteria associated with the method selection must have influences between the criteria clusters. Another significant finding is that the suggested model is more mirroring the relative of how the assortment criteria sway the chosen procedure and at the identical time what is more important for a method amidst the selection criteria.

A methodology encompassing interdependencies between nanoparticle groundwork method assortment criteria is developed and offered in this paper. An algorithm having six steps was drawn. Number of factors disagrees with ones’ workplace characteristics. Therefore, there are possibilities to boost the number of factors in preparation method assortment form up to the limit permitted. Objectivity of nanoparticle groundwork method assortment is emphasized in this paper. The foremost clarification to be made is if a factor befits to be a criterion or not. It is significant and even very absolutely vital because, while the method evolved gives the possibility to advance objectively making the right decisions, keeping the factors in the model to stand ahead. In a method selection procedure founded on factors that does not characterize the job, incorrect choices may happen and the incorrect choice could lead to the eventual product having to be formulated and evolved afresh and therefore factors have to be properly selected exclusively for every part, workplace, and position.

## Conclusion

Multi-Criteria decision-making is a well-known branch of decision making. ANP is one of the most-used methods for decision-making in the publications. In our study, we think about the difficulty, “method assortment” for the preparation of nanoparticle. For today’s growing and competitive pharmaceutical market, “method assortment” is so significant for each of a formulation when abounding of procedures are available. In this study, we utilized ANP method and Super decision software for nanoparticle formulation method selection. With the outcomes, it can be said that choosing alternate M5 to choose as the most reasonable and the most feasible outcome. Then Alternative M1, M7, M6, M3, M2 and M4 are feasible respectively. Finally, this paper supplies an explanatory evaluation of an analytical approach for decision making through a modeling method that has not been completely discovered by researchers or practitioners in diverse fields. For the future study, the difficulty can be explained by other MCDM methods, and the solutions can be compared. Also ANP with fuzzy numbers can be utilized for nanoparticle preparation method selection process, and intelligent programs to assess solutions automatically can be developed.

## Abbreviations

$: Dollar; NDDS: Novel drug delivery system; MCDM: Multi criteria decision making; MODM: Multi objective decision making; MADM: Multi attribute decision making; AHP: Analytic hierarchy process; ANP: Analytic network process; CRs: Criteria; ALTs: Alternatives.

## Competing interests

No competing interests.

## Authors' contributions

SS provided inputs to the study design, helped in data analysis and interpretation, and did final editing. RV reviewed literature, data analysis and helped in interpreting data and writing the manuscript. All authors approved and read the final manuscript.

## Authors' information

RV: Ph.D Research scholar, Department of Pharmacy, Annamalai University, Annamalai nagar 608002, Tamilnadu, India. SS: Assistant Professor, Department of Pharmacy, Annamalai University, Annamalai nagar 608002, Tamilnadu, India.
